# Trends and patterns of insecticide susceptibility of *Anopheles gambiae* between 2015 and 2020: implications for malaria vector control interventions in Ghana

**DOI:** 10.1186/s12936-025-05733-8

**Published:** 2025-12-11

**Authors:** Andy Asafu-Adjaye, Joseph Chabi, Keziah Malm, Otubea Owusu Akrofi, Sylvester Coleman, Dominic B. Dery, Yemane Yihdego, Kwadwo Kyereme Frempong, Sellase Pi-Bansa, Michelle Ayuritolya Asigbaase, Rebecca Pwalia, Joannitta Joannides, Kojo Yirenkyi Sakyi, Osei Kwaku Akuoko, Joseph Harold Nyarko Osei, Duncan K. Athinya, Aba Baffoe-Wilmot, Constance Bart-Plange, Daniel Adjei Boakye, Samuel Kweku Dadzie

**Affiliations:** 1 Vector Research Group, Department of Parasitology, Noguchi Memorial Institute for Medical Research, College of Health Sciences, University of Ghana, Legon, Ghana; 2 National Malaria Elimination Programme (Formerly National Malaria Control Programme), Ghana Health Service, Accra, Ghana; 3 Department of Vector Biology, Liverpool School of Tropical Medicine, Liverpool, UK; 4 USAID/PMI, No. 24 Fourth Circular Road, Cantonments, P.O. Box 1630, Accra, Ghana; 5 Vestergaard Frandsen (E.A) Ltd, Nairobi, Kenya; 6 Biomedical and Public Health Research Unit, Water Research Institute, Council for Scientific and Industrial Research, Accra, Ghana; 7 Centre for Research in Applied Biology, University of Energy and Natural Resources, P. O. Box 214, Sunyani, Ghana; 8 Abt Associates, PMI VectorLink Project, House 59a; Dade St., Labone, Accra, Ghana

**Keywords:** *Anopheles gambiae*, Susceptibility, Insecticide resistance, Knockdown resistance, L1014F, G119S, Malaria, Ghana

## Abstract

**Background:**

The distribution and use of insecticide treated nets (ITNs) and indoor residual spraying (IRS) are the two main insecticide-based tools that have been scaled up in Ghana. Although insecticide resistance to different insecticides have been reported in parts of Ghana, there is little information on the status and extent of resistance in malaria vectors across different areas in the country. There was, therefore, a need to generate annual geographically specific insecticide resistance data to assist in the development of insecticide resistance management strategies in Ghana. This study presents five years data on monitoring of four classes of insecticides.

**Methods:**

Standard World Health Organization (WHO) insecticide susceptibility tube assays were carried out annually on adult female *Anopheles gambiae* reared from immature stages in 30 sentinel sites across Ghana each year from 2015 to 2020. Insecticide papers impregnated with seven insecticides in four classes were used for assays in the selected sites. PCR was performed to identify mosquito species and detect knock down resistance, *kdr-west* (L1014F) and acetylcholinesterase, *ace-1* G119S gene mutations in forty randomly selected mosquitoes per site.

**Results:**

High resistance to pyrethroids and DDT was detected in all the sites with a decrease in susceptibility over the years. The susceptibility of carbamates and organophosphates reduced in all the sites over the 5 years. *Anopheles gambiae *sensu stricto (*s.s.)* and *Anopheles coluzzii* represented the two-main species of the complex living in sympatry mostly in the southern sentinel sites, with *Anopheles arabiensis* in smaller numbers in the northern Sahelian sites. The target site resistance frequency was significantly higher for the L1014F compared to the G119S with variations across the sites.

**Conclusion:**

Findings showed that resistance to deltamethrin, permethrin (pyrethroids), DDT (organochlorine) malathion and pirimiphos-methyl (organophosphates) and bendiocarb and propoxur (carbamates) was widespread in Ghana. There is high frequency of L1014F alleles (*kdr-west)* in all the sites with marginal increase for G119S *ace-1* mutations. This highlights the need for continuous monitoring of the insecticide resistance of *An. gambiae *sensu lato in the country to ensure informed decision-making on resistance management strategies and choice of insecticides for malaria vector control in Ghana.

## Background

The continued threat of malaria in many parts of the world has underscored the critical importance of vector control as a preventive strategy [1]. Central to this strategy is the use of insecticides. The renewed interest in the use of insecticides in malaria control is based on the observations that insecticide-treated materials (ITM) can reduce malaria transmission and morbidity substantially [2–6]. However, the efficacy of these interventions depends mainly on the susceptibility of vector species to the insecticides of choice. The effectiveness of insecticide-based interventions, such as the mass distribution and use of insecticide-treated bed nets (ITNs), and indoor residual spraying (IRS), is threatened by the emerging resistance of mosquito vectors, particularly in the predominant vector *Anopheles gambiae*, across sub-Saharan Africa [7–9]. There are concerns that the slow gains in malaria control since 2015 could in-part be attributed to pyrethroid resistance [10].

Recent data from the World Health Organization (WHO) Global Observatory on Health Research and Development suggests that 28 vector control products are presently under research and development to address the biological threat of insecticide resistance [11]. While there have been advances in ITNs with synergists or non-pyrethroid active ingredients such as piperonyl butoxide (PBO), pyriproxyfen or chlorfenapyr based nets to address resistance, a dependency on pyrethroids persists, due to their safety, rapid knockdown effect and relatively lower cost. This reveals a vulnerability, where rising resistance to pyrethroids threatens not just the traditional but also the emergent, advanced classes of ITNs [10], necessitating urgent strategic revisions to maintain the efficacy of malaria prevention tools.

The distribution and use of ITNs and IRS have been promoted as primary vector control tools in Ghana, where malaria remains endemic [12]. While the country’s National Malaria Elimination Programme (NMEP) restricted IRS implementation to areas of high malaria endemicity, ITNs remain the main vector control intervention across all regions in Ghana [13]. In 2018, the mass ITN distribution campaign averaged an 89% coverage rate across 194 districts in nine out of ten regions, only exempting districts where IRS was implemented. However, reports of *An. gambiae *sensu lato (*s.l.*) resistance to pyrethroids and other insecticide classes are emerging from parts of the country [8, 9]. This resistance phenomenon obscures the prospects of achieving success with the current insecticide-based vector control tools in disease prevention.

An assessment of insecticide resistance distribution maps for malaria vectors in Africa lacks such detailed and longitudinal trends for data from Ghana, which are very useful in the understanding of resistance patterns [14]. These maps offer a comprehensive visualization of areas where mosquitoes exhibit resistance to standard insecticides. Such a visual tool aids public health officials, policymakers, and researchers in allocating resources more effectively, tailoring malaria control strategies, monitoring resistance patterns, guiding further research on resistance mechanisms, and raising awareness among key stakeholders regarding the gravity and geographic distribution of resistance. Understanding resistance mechanisms, species distribution, and resistance profiles of vector populations [15] is crucial to addressing insecticide resistance and tailoring malaria interventions appropriately. Factors like geographical diversity in resistance patterns and the role of specific resistance alleles need to be factored into strategic planning for new interventions that address insecticide resistance [16].

To help generate data on the status of insecticide susceptibility of *An. gambiae* across the country, an annual insecticide resistance monitoring plan was instituted in 2015 [17]. This paper reports on data collected across 30 sentinel sites in Ghana between 2015 and 2020 highlighting the spread of insecticide resistance across the different regions of Ghana to four classes of insecticides (pyrethroids, carbamates, organochlorines and organophosphates) and its implications on malaria vector control decision making.

## Methods

### Sentinel sites

**Fig. 1:**
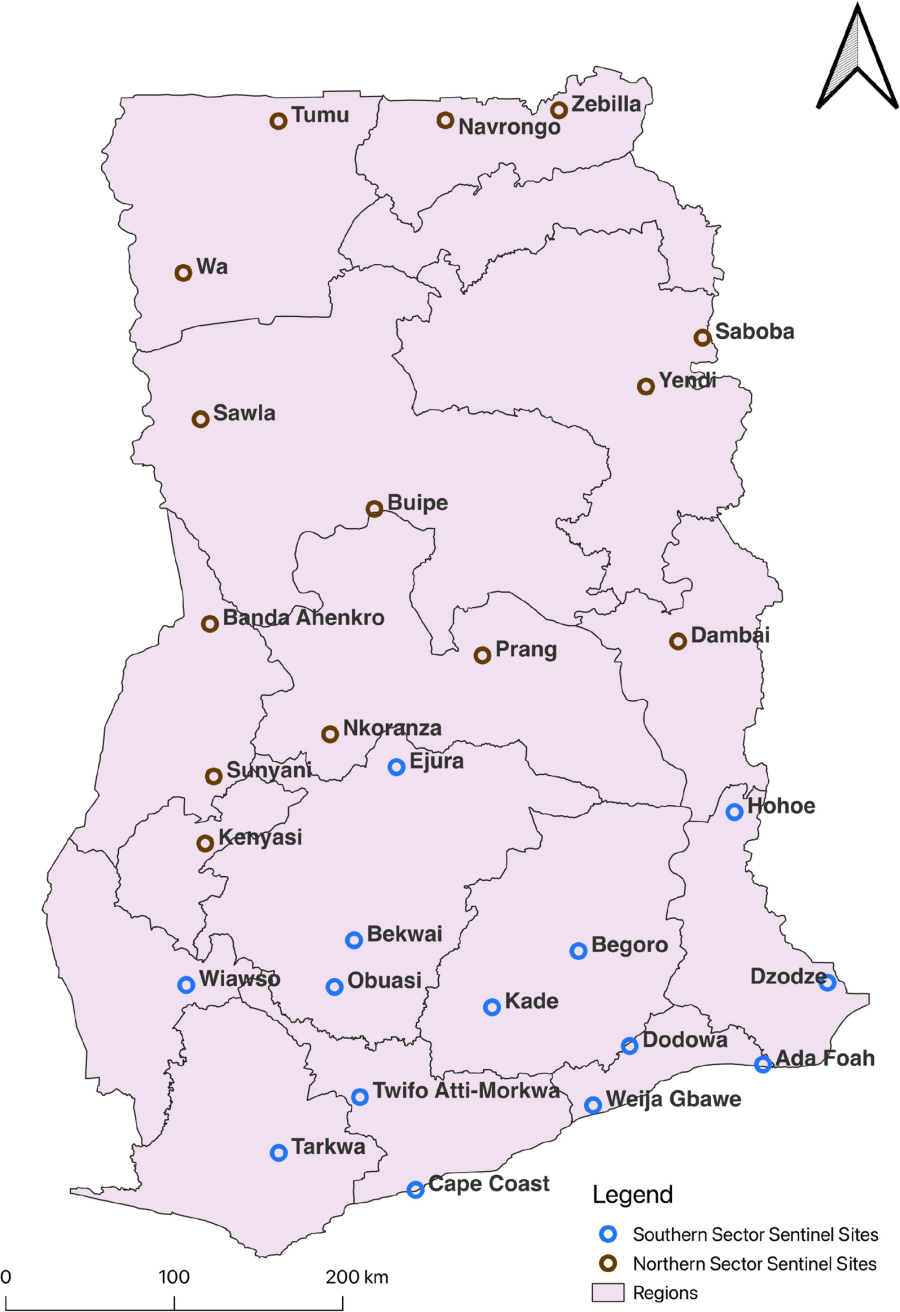
Map showing the 30 sentinel sites where the insecticide resistance surveillance was carried out across Ghana (2015–2020)

Two sites in each of the ten administrative regions of Ghana were purposely selected to serve as sentinel sites for monitoring insecticide resistance in 2015 (20 sites initially). Ten additional sites were selected due to the creation of six new regions in 2019. For the purposes of sampling coinciding with the rainfall pattern in each sentinel site, the country was divided into southern and northern sectors (Fig. 1). The sampling strategy included annual insecticide susceptibility testing and resistant allele genotyping (*kdr-west,* L1014F and *ace-1*, G119S) performed in each site between March to July in the southern sector and August to November in the northern sector. The sentinel sites were selected to be representative of the entire country covering the different ecological areas and based on information on agricultural practices, mining and other activities that could lead to the development of insecticide resistance. These sites ranged from urban, semi urban to rural communities. The selection also included areas where ITNs/IRS interventions were deployed by the National Malaria Elimination Programme (NMEP), as well as areas where there was increased agricultural practices and high use of pesticides.

### Mosquito sampling and insecticide susceptibility bioassays

Annually, mosquito larvae and pupae were collected from larval habitats in communities around each of the sentinel sites. Collections were performed around the peak rainy season, within a range of 5–10 km distance around each sentinel site. The larvae were reared to adults in each site in a temporary set-up insectary and *An. gambiae s.l.* morphologically identified using the identification keys of Gilles and de Meillon [18] prior to testing. The WHO susceptibility tube bioassay procedure [19] was used to assess the susceptibility of *An. gambiae* to over seven different insecticides representing four classes. These include pyrethroids (0.05% deltamethrin, 0.05% alpha-cypermethrin and 0.75% permethrin); organophosphates (0.25% pirimiphos-methyl, and 5.0% malathion)*;* carbamates (0.10% bendiocarb, 0.1% propoxur) and organochlorine (4% DDT). Non-blood fed female *An. gambiae* mosquitoes aged 3–5 days were selected and tested against the different insecticides. All the mosquitoes tested were kept in silica gel separated into those dead and alive for each insecticide and sent to the laboratory for further analyses. The average temperature (25–28 °C) and relative humidity (80 −85%) were maintained during the exposure and holding periods were recorded in all the sites.

### Mosquito DNA extraction and vector species identification

Annually, about 40 mosquitoes were randomly selected from each site among the mosquitoes preserved from the WHO susceptibility testing (10 per class of insecticide i.e. 5 alive & 5 dead from the bioassay). DNA was extraction from each mosquito using a slightly modified protocol by de La Cruz- Ramos et al. [20] as follows: Each whole mosquito was ground in 200 µl Cetyl Trimethyl Ammonium Bromide (CTAB) solution containing 2% CTAB, 100 mM Tris–HCl (pH 8.0), 10 mM EDTA, 1.4 M NaCl using a sterile plastic pestle, in a sterile 1.5 ml tube. The homogenate was incubated at 65 °C for 5 min and 200 µl chloroform added to each sample. This was mixed gently and then centrifuged at 12,000 rpm for 5 min. The supernatant was transferred into a new sterile tube and 200 µl isopropanol added to each sample, mixed and then centrifuged again at 12,000 rpm for 5 min. The isopropanol (supernatant) was discarded and then blotted on paper towel to remove excess liquid. Then 200 µl of 70% ethanol was added to each sample and centrifuged at 12,000 rpm for 5 min. The ethanol was discarded and blotted on paper towel to remove excess ethanol overnight. DNA pellets were reconstituted in a 20 µl nuclease free water and warned at 55 °C to dissolve the pellet. One microliter of a 1/10 dilutions of the DNA solution was used for the polymerase chain reaction (PCR). Members of the *An. gambiae* complex were identified to sibling species using the protocols described by Scott et al. [21] and Santolamazza et al*.* [22] from all the 40 samples selected per site.

### L1014F and G119S genotype analysis

The conventional PCR technique described by Martinez Torres et al. [23] was used to detect the presence and frequency of West Africa knockdown resistance gene (*kdr-w, L1014F)* and the protocol of Weill et al. [24] for the detection of the acetylcholinesterase (*ace-1, G119S)* mutations in the 40 selected *An. gambiae* mosquitoes from each site.

### Statistical analysis

WHO susceptibility criteria were followed to estimate the susceptibility status of the vector per site; mortalities of 98–100%, 90–97% and less than 90% denote susceptible, suspected resistance and resistance populations respectively [19]. Abbott’s formula was used to correct the mortality against the insecticide when the mortality (M) of the control tubes was (5% < M < 20%) [25]. Mortality values over the years were visualized and compared through paired years and insecticides using Wilcoxon signed-rank test in R Version 4.1.2. Hardy–Weinberg formula was used to determine the resistance allele frequency among the populations tested.

## Results

### Spatio-temporal trends in phenotypic resistance in *An. gambiae*

**Fig. 2:**
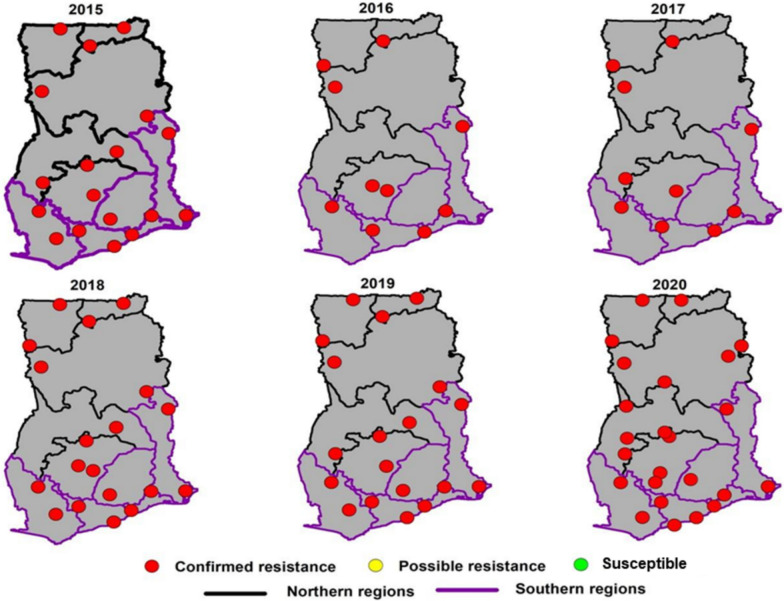
Spatial distribution of average pyrethroid resistance in regions of Ghana from 2015 to 2020

**Fig. 3:**
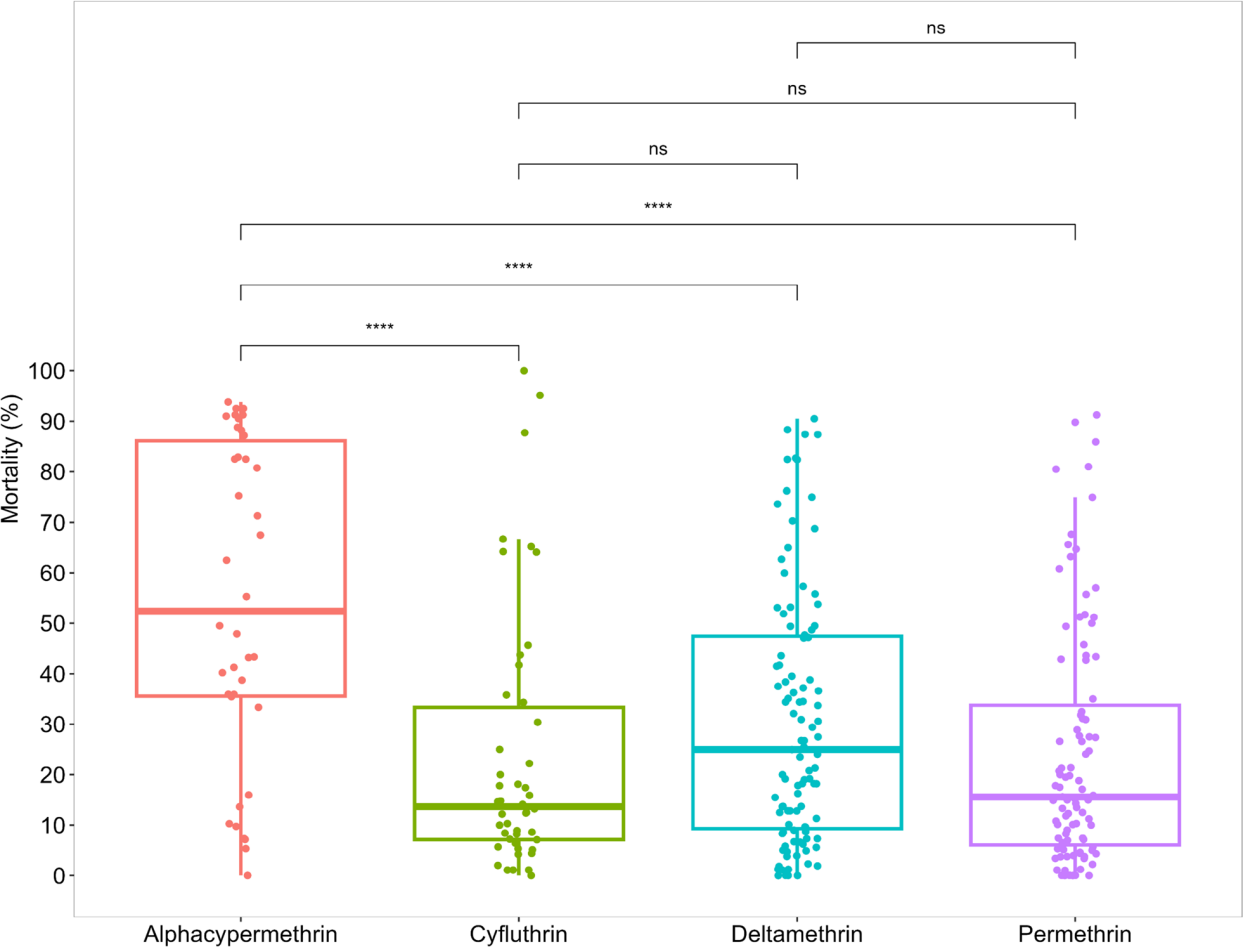
Comparative analysis of resistance of the various pyrethroids tested in the year 2015 versus the year 2020. Asterisk (*) indicates statistically significant difference (p < 0.05), ns indicates no statistically significant difference

**Fig. 4:**
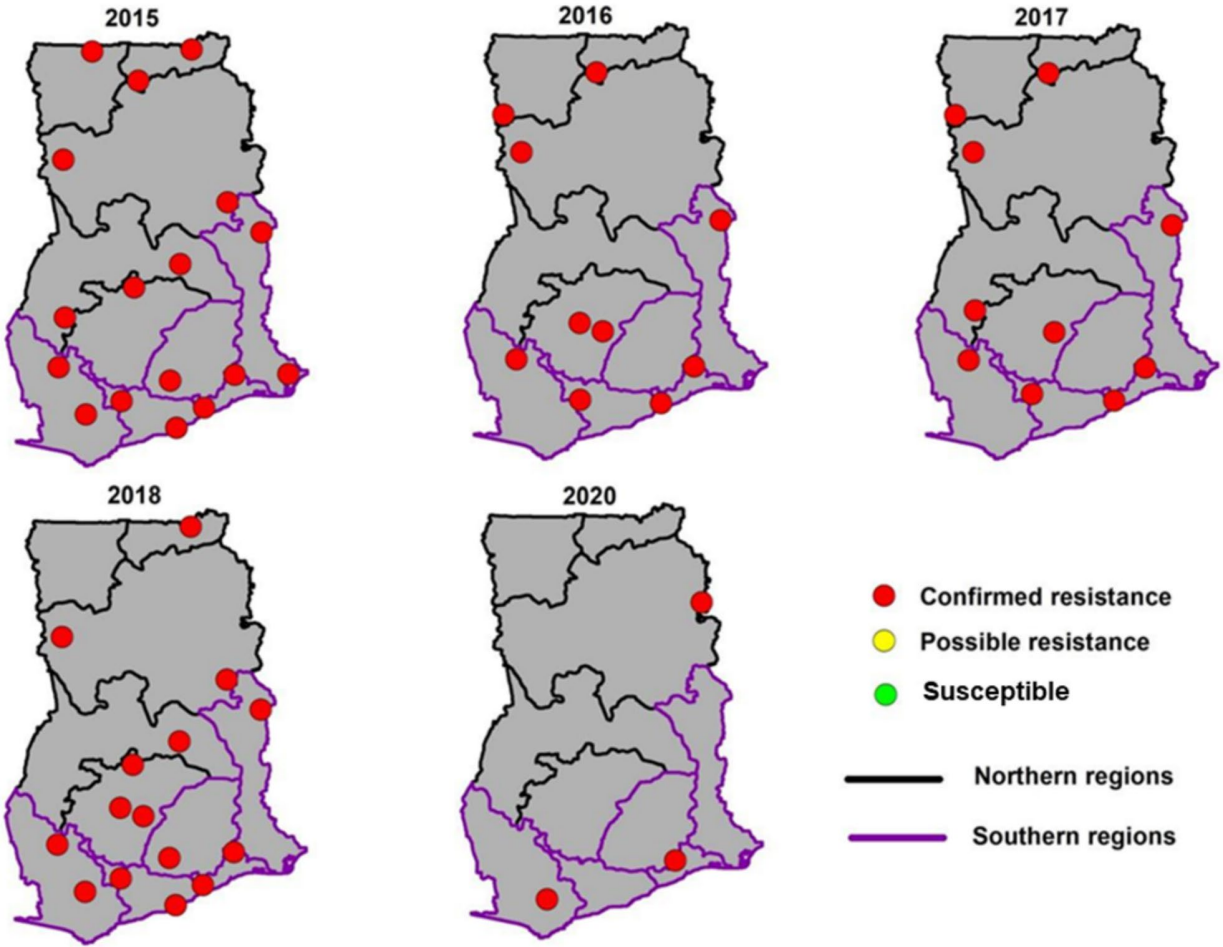
Spatial distribution of resistance to organochlorine (DDT) in Ghana from 2015 to 2020

**Fig. 5:**
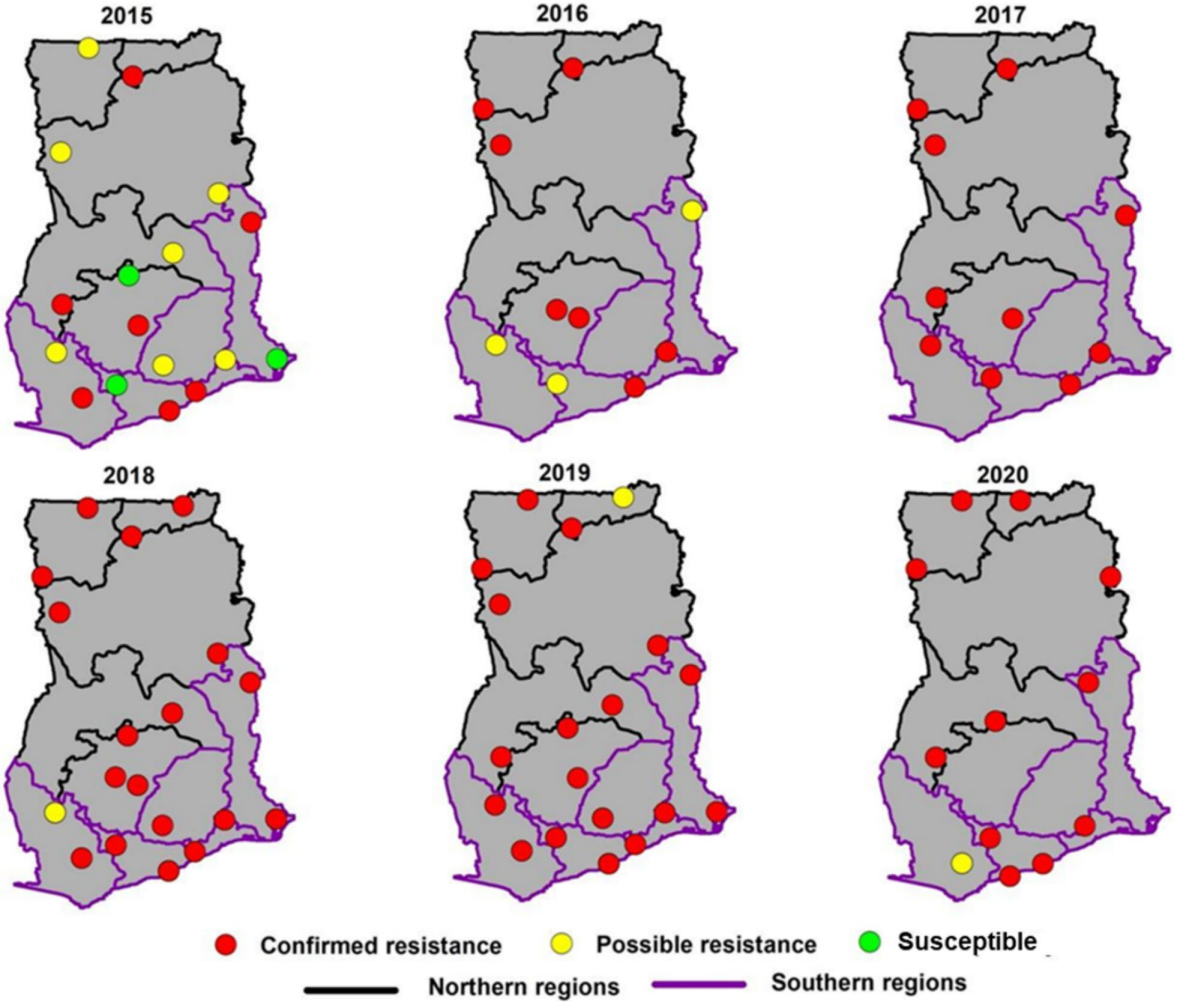
Spatial distribution of resistance to carbamates in Ghana from 2015 to 2020

**Fig. 6:**
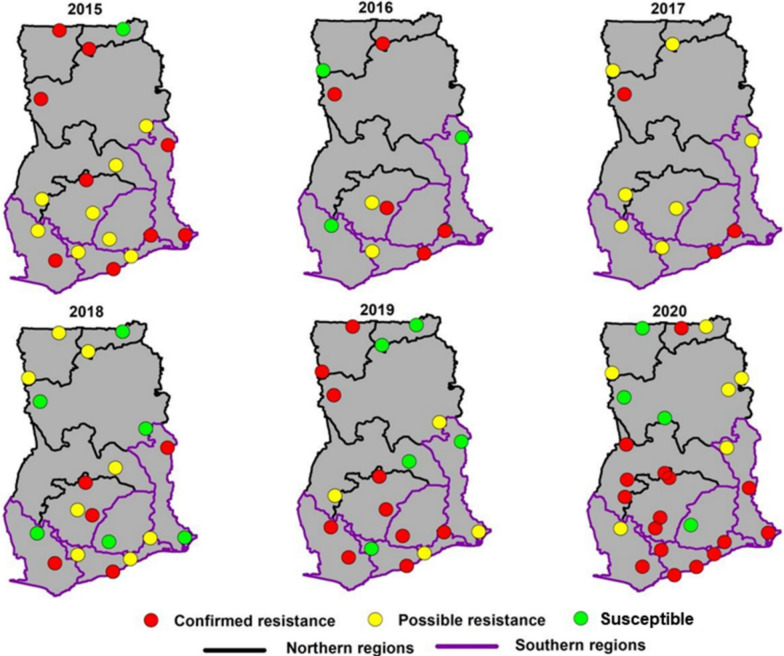
Spatial distribution of resistance to organophosphates in regions of Ghana

Figures 2, 3, 4, 5, 6 illustrate the temporal trends in resistance of *An. gambiae s.l.* (the predominant vector species in Ghana) to the four main insecticide classes (pyrethroids, organochlorines, carbamates and organophosphates respectively) tested across different sites between 2015 and 2020. The analysis of mortality rates demonstrated distinct trends among different insecticide classes.

### Pyrethroids

**Fig. 7:**
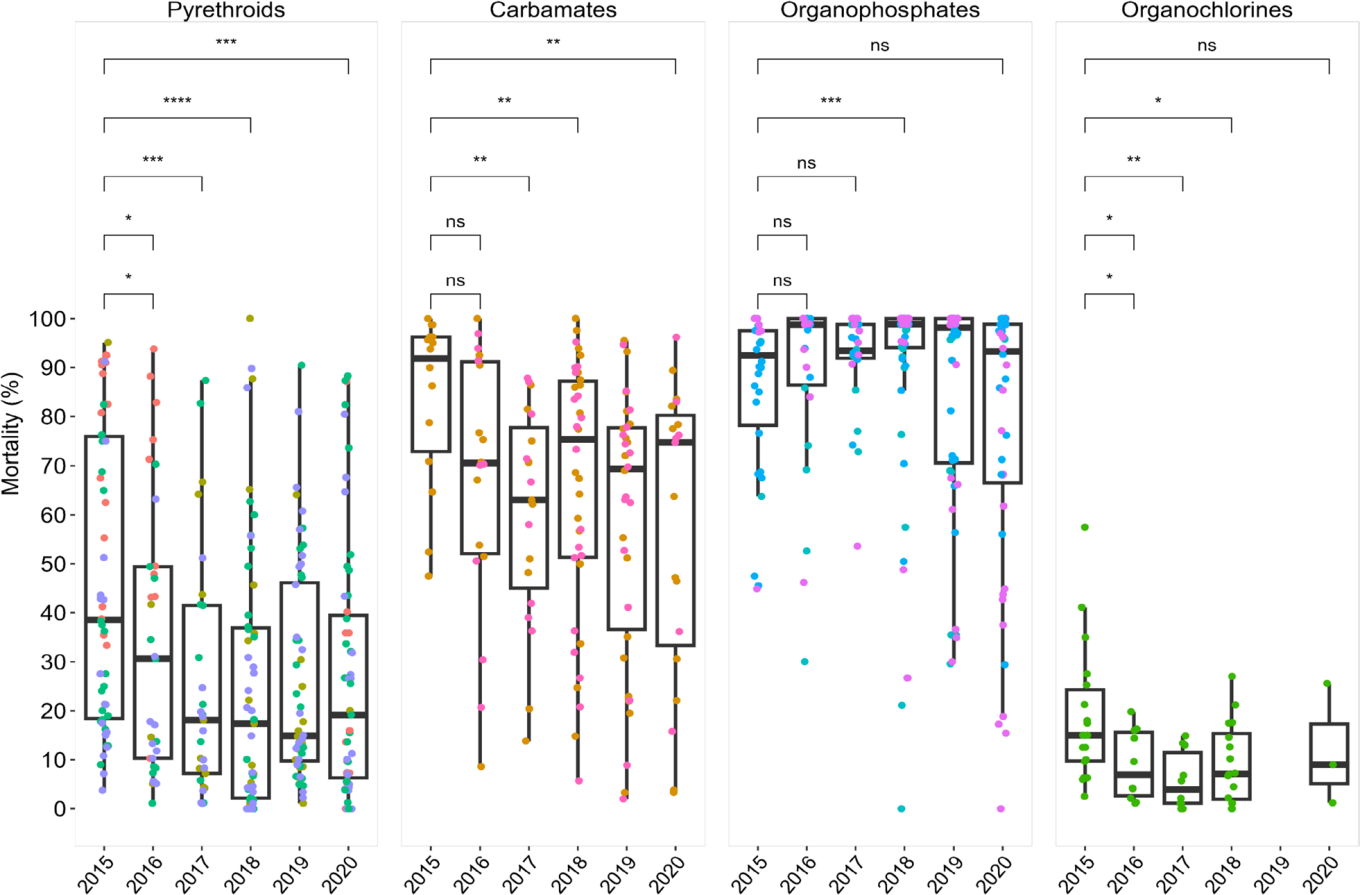
Trends in insecticide resistance levels in *Anopheles gambiae s.l*. over 5 years (2015 to 2020) in Ghana. Organochlorines were not tested for the year 2019. *Note:* asterisk (*) indicates statistically significant difference (p < 0.05), ns indicates no statistically significant difference

Throughout 2015–2020, pyrethroid resistance was evident in all 30 study sites with mosquito mortalities remaining below the 90% threshold (Fig. 2). The median mortalities among mosquitoes exposed to the different pyrethroids (alphacypermethrin, cyfluthrin, deltamethrin and permethrin) ranged between 15 to 55%, with moralities as low as 0–10% and as high as 90–100%. Alphacypermethrin showed the highest median mortality, and this was statistically significantly different from the other pyrethroid insecticides (*P* < 0.0001). The lowest were cyfluthrin and permethrin which had comparable median mortalities (Fig. 3). Mortalities for alphacypermethrin and deltamethrin were more variable than cyfluthrin and permethrin as shown in the interquartile range (Fig. 3). In terms of the percentage mortality, the years 2018 and 2019 showed lower median mortality rates compared to the 2015 and 2020, and a general decline in susceptibility of the vectors to pyrethroids observed from 2015 to 2020 (Fig. 7).

### Organochlorines

DDT was the only organochlorine tested. It showed consistently low mortality rates over the years, staying mostly below the 90% threshold (Fig. 3). Median mortality rates in 2016 and 2017 were lower compared to subsequent years. From 2017 onwards, a marginal increase in the mortality rate was observed. The interquartile range indicated stable data distribution over the years.

### Carbamates

Mortality rates to carbamates were higher compared to pyrethroids or organochlorines with mortality in most sites ≥ 90% in 2015. Over five years, an increase in confirmed resistance to carbamates was observed across all 16 regions. Susceptible populations, as represented by green dots, diminished over time, and absent in 2020 (Fig. 4). Possible resistance, though sporadic, was widespread in the initial year (2015), decreased in 2016 and then became less prevalent, with only isolated instances from 2018 to 2020. The decrease in mortality over the years was statistically significant relative to 2015 except for the year 2016 (Fig. 6).

### Organophosphates

The temporal patterns of resistance to organophosphates in all 16 regions from 2015 to 2020 fluctuated year-on-year and among the sites. The initial years showed a balance between confirmed resistance, possible resistance, and susceptible. Bioassay with organophosphates displayed a mortality ranging from 60 to 100%. The median of multiple data points in the organophosphates group of insecticides remained above the 90% threshold of mosquito mortality. However, by 2020, the regions located in the South experienced an increase in confirmed resistance. The Northern sectors, on the other hand, showed more variability, with an increase in susceptibility in 2018. When the efficacy of the different organophosphates was compared across all sites, it was observed that mosquito mortalities to pirimiphos-methyl remained consistently between 90 and 100%. There was a drop in median mortality rate in 2019, but it appeared to rebound in 2020.

### Characterization of the target site mutations

**Fig. 8:**
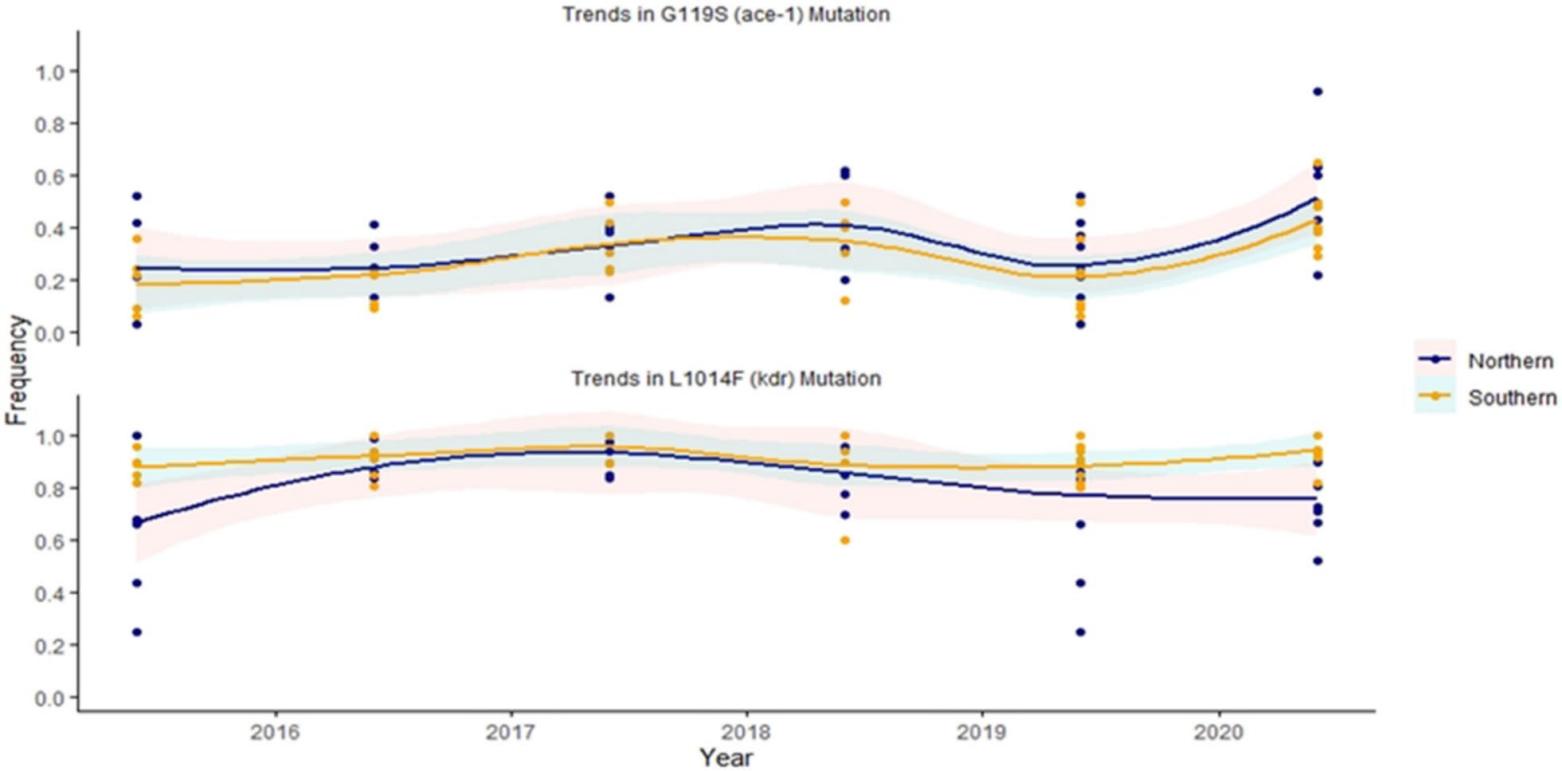
Temporal trends of G119S (*ace-1*) and L1014F (*kdr-west*) mutation frequencies in mosquito populations of northern and southern sites in Ghana (2016–2020) revealed by LOESS Curves. The shaded regions around each curve represent the standard deviations, indicating the variability in the observed data around the fitted trend

The temporal patterns of G119S (*ace-1)* and L1014F (*kdr-w)* mutations in the mosquito populations in both the Northern and Southern Sectors of Ghana between 2015 and 2020 were distinct (Fig. 8). The Locally Weighted Scatter-plot Smoother (LOESS) curves depicting the *ace-1* mutation, associated with resistance to organophosphates and carbamates, show contrasting patterns for the two operational Sectors. Frequencies of the *ace-1* mutation remained lower compared to *kdr-w* mutation across all the sites. In the northern sector, there was a 10–15% rise in the frequency of *kdr-w* mutation in the mosquito populations tested in 2017, followed by a steady decline until 2020. Conversely, the frequency in mosquito populations in the southern sector remained high throughout the period. By 2020, this frequency increased to 0.95 (Fig. 8). There was about 10% increase in frequency of the *ace-1* resistant alleles from 2019 to 2020 in both northern and southern sectors. Throughout the period, *An. gambiae* populations in the northern sector consistently showed a relatively higher frequency of the *ace-1* (G119S) mutation compared to mosquito populations in southern Ghana (Fig. 8). On the other hand, the trend is distinct for *L1014F* (*kdr*-*w*) mutation which confers resistance to pyrethroids and organochlorines via amino acid replacements in the voltage-gated sodium channel (VGSC). The *kdr-w* mutation appeared to be almost fixed in most of the sites tested with frequencies ranging between 0.8 and 1.0, with slight variations. In the northern sector about 70% of the sites recorded *kdr-w* resistance allele frequencies between 0.8 and 1.0. The lowest frequencies were recorded around 0.25 and 0.44 (Fig. 8). However, generally the frequency for this mutation showed mild fluctuations from 2015 through 2018, culminating in a slight decline between 2019 and 2020. For the southern sector, the mosquitoes maintained a more consistent allele frequency for the *kdr-w* mutation across the five years, with a minor peak. In the earlier years of the study, the southern sectors generally showed a higher allele frequency for the *kdr-w (L1014F)* mutation relative to the northern sector, but this disparity slightly lessened by 2020.

### *Anopheles gambiae* s.l. species composition

**Fig. 9:**
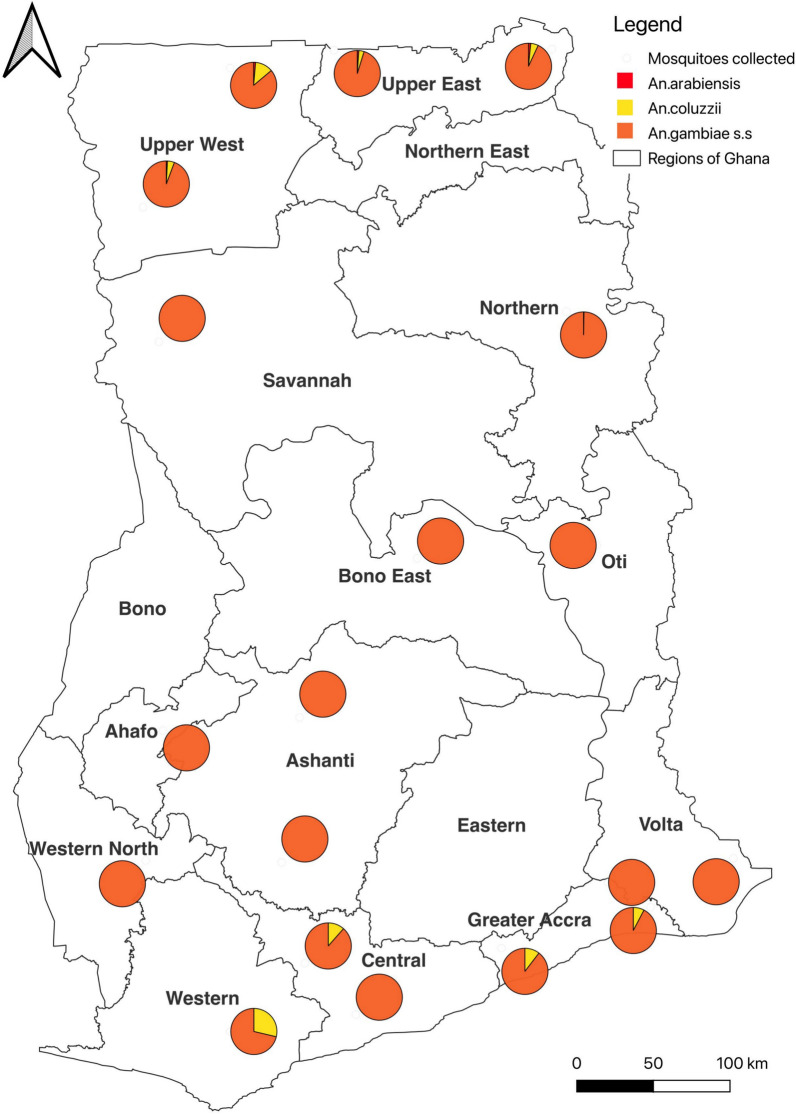
Distribution and relative abundance of *Anopheles* mosquito species across the sentinel sites over the period (combined over the period). *An. gambiae s.s* and *An. coluzzii* represented the two-main species of the complex living in sympatry especially in the southern sentinel sites (where *An. coluzzii* numbers were relatively higher than observed in the northern area), with *An. arabiensis* in smaller numbers in some sites within the northern Sahelian area

Based on cumulative analysis of species obtained over the five-year period, *An. gambiae s.s.* and *Anopheles coluzzii* represented the two-main species of the complex in the southern sites while *Anopheles arabiensis* was found in some northern sites (Fig. 9). *Anopheles gambiae s.s.* was the primary species found in all the southern sites with > 90% composition in most of the southern sites. Further identification of the *An. gambiae s.s*. showed *An. gambiae s.s* co-existing in sympatry with *An. coluzzii* in almost all sites especially in the southern humid sites within southern Ghana. While in the middle belt of Ghana it is mainly *An. gambiae s.s.* (100.0%), the southernmost part of Ghana had both *An. gambiae s.s*. (71.3–100.0%) and *An. coluzzii* (7.7–28.7%), and the northern parts had *An. arabiensis* (0.0–1.6%), *An. coluzzii* (0.3–12.5%), and *An. gambiae s.s*. (86.1–99.7%) (Fig. 9).

## Discussion

Ghana is aiming to move into the malaria elimination phase and has developed a new strategic plan to guide the process [26]. Currently, the use of LLINs and the IRS are the primary interventions for malaria vector control. This paper presents the first national baseline insecticide profile in Ghana and a five-year continuous data on resistance monitoring of *An. gambiae*, the main malaria vector in Ghana.

The study results suggest insecticide resistance in *An. gambiae s.l.* has progressively increased over the period. This increase is seen not only in the frequency of resistance phenotypes but also in the geographical spread in both northern and southern sites of Ghana. In addition to the confirmed role of oxidases in pyrethroid resistance previously reported [17], there was an increased frequency in target site mutations for L1014F and G119S (*ace-1*) from the data spanning 2015 to 2020. The marginal decline in L1014F frequency in the northern mosquitoes as opposed to the continually high frequency in the southern mosquito populations suggests a possible change in selection pressure or other ecological factors influencing the mosquito populations in the northern sector. The decision to distribute ITNs only to districts without IRS may have played a significant role. While all districts in the southern sector continued to receive ITNs through either mass campaigns, IRS with non-pyrethroid insecticides was implemented in 22 districts in the north which did not receive ITNs. In the southern sites, the increasing trend in L1014F might be attributed to the continuous distribution of pyrethroid-only bed nets across all districts. Another potential contributor to this trend, especially in the southern sector where agricultural farming is prevalent, could be the agricultural application of pyrethroids, organophosphates, and carbamates. In a recent study on pesticide application practices in four regions of Ghana (Ashanti, Central, Western, and Western North), Boateng et al*.* [27] identified bifenthrin, etofenprox and alphacypermethrin (pyrethroids) as the most commonly used active ingredient used by Ghanaian cocoa farmers (43%) in the sampled regions. Similarly study in northern Ghana [28] found that lambda-cyhalothrin was the most used insecticide (50%) by farmers from 20 communities in the Tolon district of the Northern sector of Ghana. The study also revealed that up to 64% of the respondents disposed of their empty pesticide containers indiscriminately. Such disposal practices are potential sources of pesticide contamination due to run-off into preferred anopheline larval habitats in agricultural farmlands [29]. Apart from the interventions and agricultural practices, these differences between northern and southern sites could also be due to the different ecological zones and the species composition of these vectors.

The increase in G119S frequency was seen in both northern and southern sectors. It is possible that extensive use of carbamate and organophosphate insecticides in residential pest control and agricultural purpose such crop farming [30, 31] or animal husbandry and livestock [32] across the different sites could in-part be driving selection for resistant vector populations. In a study in Dormaa West District, Fosu-Mensah et al. [31] reported the presence of 13 organophosphorus pesticide residues in soils and drinking water in cocoa-producing areas of Ghana due to increased pesticide use to enhance productivity. Some of the pesticides reported in this study include chlorpyrifos, diazinon, profenofos, and pirimiphos-methyl. The use of carbamate pesticides such as carbofuran along with chlorpyrifos (an organophosphate pesticide) for vegetable farming have also been reported in other parts of the country [33].

Previous studies across Africa have found that prolonged use of pesticides in farming can lead to mosquito populations exposed to sub lethal concentrations of these active ingredients. Such encounters can select for the survival of mosquitoes resistant to these chemicals which in turn could influence the observed resistance patterns [34–37]. Nkya et al. [29] found that mosquitoes with prior exposure to sub-lethal doses of pesticides or other pollutants at the larval stages display higher tolerance to insecticides at the adult stage, as observed in various locations throughout Africa. However, without concrete data on similar practices from study sites in Ghana, drawing definitive conclusions remain challenging to directly relate these observations to farming practices.

Even though resistance to other insecticides increased, it was observed that the mosquito populations tested remained largely susceptible to pirimiphos-methyl up until 2019 after which possible resistance then emerged in most sites (Figs. 6 and 7). The two main IRS programs funded by the U.S. President’s Malaria Initiative (PMI) and the Global Fund continued to use pirimiphos-methyl until 2019, when insecticides were switched [37] due to resistance. In addition to the impact of organophosphate and carbamate based pesticides used for domestic and agrarian purposes in driving resistance, there is data that indicate how insecticide based interventions, have contributed to the spread and intensity of pyrethroid resistance across sub-Saharan Africa [38, 39]. It is therefore likely that the increasing number of districts that were sprayed with Actellic 300CS (a long-acting formulation of pirimiphos-methyl) between 2015 and 2019 in the northern sector might in-part account for the higher *ace-1* frequency compared to southern Ghana. During the study period the PMI sprayed an average of 300,000 structures annually in northern and northeast regions of Ghana whilst Global Fund also sprayed an average of 1,0000,000 structures annually in Upper West, select districts in Upper East and Ashanti regions of Ghana [37]. The slight dip in the frequency of *ace-1* alleles in 2019 might be because of the introduction of neonicotinoid-based insecticides (Sumishield 50WG and Fludora Fusion) for IRS by both PMI- and Global Fund- funded IRS programmes [37].

It has been argued that despite developing resistance, mosquitoes exposed to ITNs experience a significant reduction in lifespan and malaria transmission potential. Even highly resistant mosquitoes showed a 50% decrease in lifespan post-exposure to ITN [40]. ITNs may continue to provide barrier protection [41]. Additionally, sublethal exposure to insecticides can influence mosquito biology, physiology, and behaviour, affecting their longevity, fertility, and feeding habits [42]. These effects may potentially influence the effectiveness of insecticide-based interventions highlighting the continued effectiveness of LLINs in malaria control strategies despite growing insecticide resistance [40, 43]. However, in certain instances, sublethal insecticide stress exposure had positive transgenerational fitness effects, leading to increased survival and enhanced competitiveness of resistant phenotypes, acting as a selective pressure for the evolution of insecticide resistance [44]. These consequences of resistance observed in mosquito vectors to widely used insecticides, such as pyrethroids and organophosphates, is causing global concern and necessitating a review of existing interventions [15, 16, 45] to maintain the gains in vector control. High levels of pyrethroid resistance could have a strong negative epidemiological impact, which could derail malaria control efforts in Africa [46]. The potency of LLINs impregnated with a single pyrethroid active ingredient (conventional nets) to offer the needed personal protection to its users, has been found to have been reduced against highly resistant mosquito populations [46]. This reduced potency could lead to increased malaria incidence due to reduced mosquito mortality and loss of community-wide protection offered by the LLINs. There is evidence to suggest that IRS and LLINs failed to achieve the desired level of control in some areas because of increased insecticide resistance in the main vectors. For instance, a malaria epidemic in 2000 in KwaZulu Natal province of South Africa was attributed to the presence of pyrethroid resistant *Anopheles funestus* in the area, because the malaria programme had switched from DDT to deltamethrin [47]. Following the re-introduction of DDT, the number of malaria cases decreased to levels lower than those recorded before the epidemic [47]. Similarly in northern Ghana, prevalence of malaria asexual parasitaemia was reported to have declined only by about 9.0% and 29.2% at the end of the high and low transmission seasons, respectively, after 2 years of IRS with alpha-cypermethrin. However, a switch in insecticides to pirimiphos-methyl (an organophosphate) led to about 57% decline in the prevalence of malaria asexual parasitaemia [48].

Other studies have also documented the operational impact of pyrethroid resistance in the main malaria vectors on the efficacy of LLIN as a vector control intervention. In a study in Senegal, Trape et al*.* [49] reported that an increase in pyrethroid resistance from 8% in 2007 to 48% in 2010, 27–30 months after the distribution of LLINs led to a resurgence in malaria incidence. In western Kenya, a resurgence in vector density and malaria parasite prevalence in two out of three sentinel sites was observed despite high ownership of ITNs [50]. This was in part attributable to increased insecticide resistance in mosquitoes. Recent findings from studies in Benin and Burkina showed that the development of resistance to pyrethroids caused by voltage-gated sodium channel (*Vgsc)* mutation have been directly linked to the failure of ITNs and IRS [39, 51].

Although malaria control failures due to increased insecticide resistance have yet to be reported in Ghana, these observations of increased resistance over the years represent a clear threat to the efficacy of insecticide-based vector control strategies. There is therefore the need to periodically monitor the trends to help develop resistance management strategies that can prevent the fixation of the genes in the mosquito vector population.

## Conclusions

Monitoring insecticide resistance in malaria vectors in Ghana is crucial for sustaining the gains made in reducing malaria burden through the deployment of effective vector control tools. It is also a critical exercise for the management of insecticide resistance. This study showed that pyrethroid and DDT resistance is high across the country whilst organophosphates (pirimiphos-methyl) were found as suitable options for IRS especially in the northern part of the country. Multiple resistance mechanisms were also involved in the sentinel sites with *kdr-w* resistant alleles almost fixed in the *An. gambiae* populations. This information is important for the NMEP in the planning and deployment of vector control tools as well as managing insecticide resistance to ensure effective malaria control in the country.

## Data Availability

No datasets were generated or analysed during the current study.

## Abbreviations

DNA: Deoxyribonucleic acid
EDTA: Ethylenediaminetetraacetic acid
IRS: Indoor residual spraying
ITM: Insecticide-treated materials
ITNs: Insecticide treatment bed nets
LLIN: Long-lasting insecticidal nets
NMEP: National malaria elimination programme
PBO: Piperonyl butoxide
PCR: Polymerase chain reaction
PMI: United States President’s Malaria Initiative
WHO: World Health Organization
